# Integrated single-cell and transcriptome sequencing analyses determines a chromatin regulator-based signature for evaluating prognosis in lung adenocarcinoma

**DOI:** 10.3389/fonc.2022.1031728

**Published:** 2022-10-17

**Authors:** Qingtong Shi, Song Han, Xiong Liu, Saijian Wang, Haitao Ma

**Affiliations:** ^1^ Department of Thoracic Surgery, The First Affiliated Hospital of Soochow University, Suzhou, China; ^2^ Department of Thoracic Surgery, The Affiliated Hospital of Yangzhou University, Yangzhou, China; ^3^ Department of Thoracic Surgery, Suzhou Science and Technology Town Hospital, Suzhou, China; ^4^ Graduate School of Dalian Medical University, Dalian, China

**Keywords:** lung adenocarcinoma, chromatin regulator, prognosis, risk signature, immune microenvironment

## Abstract

**Background:**

Accumulating evidence has highlighted the significance of chromatin regulator (CR) in pathogenesis and progression of cancer. However, the prognostic role of CRs in LUAD remains obscure. We aim to detect the prognostic value of CRs in LUAD and create favorable signature for assessing prognosis and clinical value of LUAD patients.

**Methods:**

The mRNA sequencing data and clinical information were obtained from TCGA and GEO databases. Gene consensus clustering analysis was utilized to determine the molecular subtype of LUAD. Cox regression methods were employed to set up the CRs-based signature (CRBS) for evaluating survival rate in LUAD. Biological function and signaling pathways were identified by KEGG and GSEA analyses. In addition, we calculated the infiltration level of immunocyte by CIBERSORT algorithm. The expressions of model hub genes were detected in LUAD cell lines by real-time polymerase chain reaction (PCR).

**Results:**

KEGG analysis suggested the CRs were mainly involved in histone modification, nuclear division and DNA modification. Consensus clustering analysis identified a novel CRs-associated subtype which divided the combined LUAD cohort into two clusters (C1 = 217 and C2 = 296). We noticed that a remarkable discrepancy in survival rate among two clusters. Then, a total of 120 differentially expressed CRs were enrolled into stepwise Cox analyses. Four hub CRs (CBX7, HMGA2, NPAS2 and PRC1) were selected to create a risk signature which could accurately forecast patient outcomes and differentiate patient risk. GSEA unearthed that mTORC1 pathway, PI3K/Akt/mTOR and p53 pathway were greatly enriched in CRBS-high cohort. Moreover, the infiltration percentages of macrophage M0, macrophage M2, resting NK cells, memory B cells, dendritic cells and mast cells were statistically significantly different in the two groups. PCR assay confirmed the differential expression of four model biomarkers.

**Conclusions:**

Altogether, our project developed a robust risk signature based on CRs and offered novel insights into individualized treatment for LUAD cases.

## Introduction

Lung cancer (LC) is the major cause of death for men and women with tumor, representing approximately 18% of all cancer deaths worldwide (1). Up to 90% of LC cases are non-small cell lung cancer (NSCLC), including both lung adenocarcinoma (LUAD) and lung squamous cell carcinoma (LUSC) histological subtypes, with LUAD occurring most frequently (2). Despite recent advances in clinical treatment, the prognosis for LUAD remains dismal, with a 5-year survival rate of only 19%. With the advent of aging and air pollution in developing countries, the incidence of LUAD remains high and early diagnosis of LUAD becomes essential (3). Unfortunately, we still have limited availability of accurate biomarkers for early diagnosis and individualized treatment of LUAD.

Tumor microenvironment (TME) is the internal environment for tumor cell production and survival, and its cellular components include resident stromal cells and recruited immunocytes in addition to tumor cells (4). TME plays an important role in the tumor growth, metastasis, angiogenesis and treatment resistance and has also a crucial impact on prognosis (5). Therefore, systematic exploration of TME is helpful to clarify the mechanism of tumor occurrence and individualized treatment.

Epigenetic modification is a reversible and heritable process of gene expression in the absence of DNA sequence changes. It is one of the critical regulatory mechanisms at the post-transcriptional level of genes by chromatin regulators (CRs), mainly including DNA methylation, histone modifications, chromatin remodeling and RNA regulation (6). CRs-mediate epigenetic modification regulates the activation of heterozygous promoters or the activity of repressors and trigger changes in gene transcription levels, resulting in cell differentiation, abnormal proliferation and tumorigenesis (7).

Numerous studies have demonstrated that CRs are tightly bound up to the patient outcomes of LC HMGA1, a chromatin remodeler, has been reported to be involved in DNA transcription, replication and repair. Saed and his colleagues have observed that HMGA1 presented higher expression in lung cancer specimens and overexpressed HMGA1 lead to dismal prognosis of LUAD (8). Moreover, HMGA1 was proved to facilitate LUAD cell proliferation and migration through GRP75-induced JNK pathway (9). EZH2, belonging to the polycomb-group (PcG) family, has been reported to be greatly overexpressed in lung specimens, and upregulation of EZH2 predicts dismal survival of NSCLC (10). Geng and his colleagues indicated EZH2 enhances the growth and metastasis of lung cell by Akt pathway (10). RAD51 is well known for its important role in homologous recombination. RAD51 has shown to be upregulated in KRAS mutant lung cancer and could regulate cell survival by enhancing DNA damage repair (11). However, the expression patterns and prognostic value of CRs in LUAD remain largely unknown.

In this academic research, we determined CRs with powerful prognostic values in LUAD and created a risk signature for clinical outcome assessment and immune status prediction of LUAD cases.

## Methods

### Data collection and processing

We obtained the RNA sequencing (RNA-seq) data of 535 LUAD patients and 59 normal controls and their corresponding clinical features from TCGA database (https://portal.gdc.cancer.gov/) to construct the prognostic signature. The transcription profiling data was downloaded from GEO dataset and was utilized as the validate set.

### Determination of differentially expressed CRs

A total of 870 CRs were retrieved from previous research (6). The gene information of all CRs summarized in **Supplementary Table S1**. The differentially expressed genes (DEGs) between normal and LUAD tumor tissues were determined using the limma R package with a criteria P value<0.05 (12). The generated DEGs and CRs gene sets were subsequently intersected to obtain differentially expressed CRs (DECRs).

### Function and pathways enrichment analyses

GO and KEGG enrichment analysis was conducted to obtain the insight into the biological functions and potential pathways of DECRs. Terms with p< 0.05 were listed and visualized using the “clusterProfiler” R packages (13).

### Integration of protein–protein interaction (PPI) network

A protein–protein interaction network (PPI) was developed and visualized using the STRING online database (https://cn.string-db.org/) and the Cytoscape (https://cytoscape.org/), respectively (14, 15). Further, the cubHubba plugin in Cytoscape software was used to filter hub genes of the PPI.

### Gene consensus cluster analysis

The consensus cluster analysis was conducted using the “ConsensusClusterPlus” R package, based on the combined LUAD cohort (16). To identify the optimal cluster value, we calculated the Delta area and the cumulative distribution function (CDF). Survival analysis was carried out to compare clinical prognoses between different subtypes using “survival” R package.

### Construction of the risk signature

Subsequently, Cox regression analyses were performed to obtain candidate CR with remarkable prognostic value. The formula was set up: Risk score = $\sum_{i=1}^{n}\left(\text{coef }\times {\text{Exp}}_{i}\right)$ . “Coef” was defined as the corresponding regression coefficient value, and “Exp” was the expression level of genes in the prognostic model. All patients were divided into low- and high-risk groups according to the median score.

### Gene set enrichment analysis (GSEA)

We performed GSEA analysis, including GO and KEGG analysis based on CR related DEGs to identify the potential biological and functional differences of different hierarchical clustering (17). A function term with an adjusted p-value<0.05 and a false discovery rate (FDR)<0.25 was considered enriched.

### Estimate of immune infiltrating status

CIBERSORT tool (https://cibersortx.stanford.edu/index.php) was employed to quantify the infiltration status of 22 types of immunocyte fractions in the two LUAD subgroups. P< 0.05 was defined as statistically significant.

### Single-cell sequencing analysis

We utilized the Seurat clustering to analyze the single-cell data acquired from the GEO databases. The UMAP dimensional reduction and the t-Distributed Stochastic Neighbor Embedding (tSNE) method, were employed to visualize the gene expression and distribution in dataset GSE131907. Next, the cells were re-clustered with the “SingleR” packages to demonstrate the feature genes of different cell types.

### Validation of the model CRs

To detect the expression pattern of a model gene at the mRNA level, GEPIA2 tool was applied. Human Protein Atlas (HPA, https://www.proteinatlas.org/) database was utilized to confirm the protein level of our model genes between LUAD and normal control (18).

### Somatic mutation and stem cell characteristics analyses

The somatic mutation data were obtained from TCGA Portal and processed to compare the tumor mutation burden (TMB) in two groups. The mRNAsi is a quantitative index reflecting cancer cells calculated based on gene profiles; The mRNAsi and TMB differences in two subgroups were compared using the independent-samples t-test.

### Cell culture

Two human LUAD cell lines (A549 and NCI-H460) and a normal human lung epithelial cell line (BEAS-2B) were purchased from American Type Culture Collection. All cell lines were cultured in RPMI 1640 medium (Sigma) containing 10% fetal bovine serum (Gibco) and 1% antibiotics (100 U/ml penicillin G and 100mg/ml streptomycin) at 37°C in a humidified chamber containing 5% CO2.

### RNA extraction and quantitative real-Time PCR

Total cell RNA was extracted by RNA isolation reagent (Takara), then reversed into cDNA by PrimeScript Mix reagent (Takara). SYBR Green Premix (Vazyme biotech) was utilized for PCR reaction system. The value of individual genes was standardized to GAPDH expression level. **Supplementary Table S2** displays primer sequences of all genes.

### Statistical analysis

All statistical data in the present project was analyzed by R version 4.0.5 and GraphPad Prism 9. The Kaplan-Meier (KM) analysis was employed to assess the prognostic value of the signature. Moreover, we plotted the receiver operating characteristic (ROC) curve over time to evaluate the prognostic efficacy of the signature.

## Results

### Characterization of chromatin regulators in LUAD

**Figure 1:**
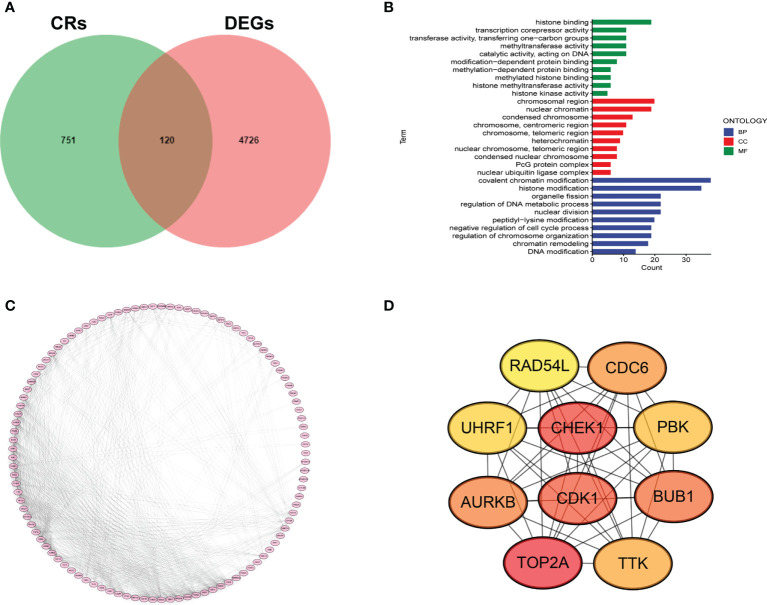
Determination of DECRs in LUAD. **(A)** Venn diagram of DECRs. **(B)** KEGG analysis of DECRs. **(C)** PPI network for 120 DECRs. **(D)** The top ten hub genes of DECRs-based PPI network.

We first collected 4846 DEGs between LUAD samples and normal cases. A total of 120 DECRs were obtained by taking the intersection of CRs and DEGs gene lists (**Figure 1A**). Then, GO analysis was employed to detect the underlying function of DECRs. The result disclosed that these genes were mainly enriched in histone modification and DNA modification (**Figure 1B**). Next, we generated a PPI network to explore the protein interaction among 120 DECRs (**Figure 1C**). Based on the MCC algorithm, the top ten hub genes were selected to set up a hub network, including CHEK1, CDK1, TOP2A, CDC6, UHRF1, AURKB, PBK, BUB1, TTK and RAD54L (**Figure 1D**).

### Chromatin regulators-based consensus cluster analysis

TCGA-LUAD and GSE14520 were combined into one LUAD cohort (n = 609). We applied consensus cluster analysis to develop a CR-related molecular subtype of LUAD.

**Figure 2:**
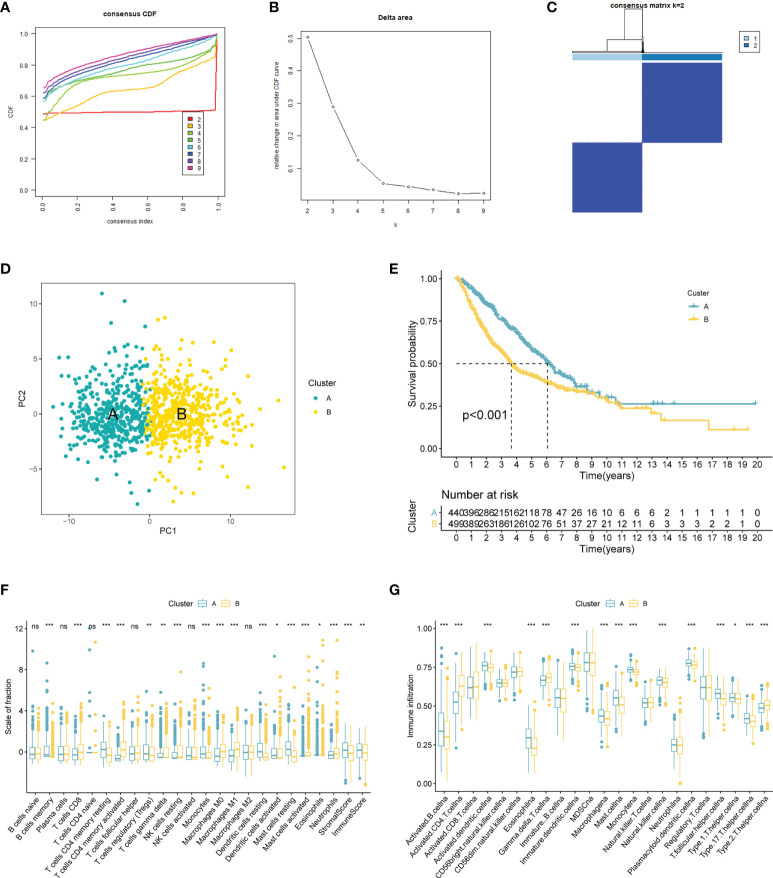
CRs-associated clustering analysis. **(A, B)** The CDF value of consensus index. **(C)** Consensus matrix for k=2. **(D)** Principal component analysis of the entire LUAD set. **(E)** The Kaplan–Meier survival analysis. **(F)** Comparison of immunocyte proportions of the CRs-based clusters. **(G)** The differences of TME score 22 the two clusters. ns, no significance; 0.05; *p < 0.05; **p < 0.01; ***p < 0.001.

The result suggested the entire dataset could be well divided into two subtypes based on the 120 DECRs when k = 2 by increasing the clustering variable (k) from 2 to 9 (**Figures 2A–C**). PCA analysis shows that DECRs can clearly distinguish two subgroups for clustering analysis (**Figure 2D**). There were remarkable discrepancies in survival rates among the two clusters (**Figure 2E**). To evaluate the TME status of two clusters, ESTIMATE algorithm was conducted. As suggested in **Figure 2F**, cluster A presented higher stromal score and immune score than that cluster B. In addition, we observed that B cells, T cells, NK cells, dendritic cells and Macrophages showed the most notable difference among the two clusters (**Figure 2G**).

### Construction of the CRBS

**Figure 3:**
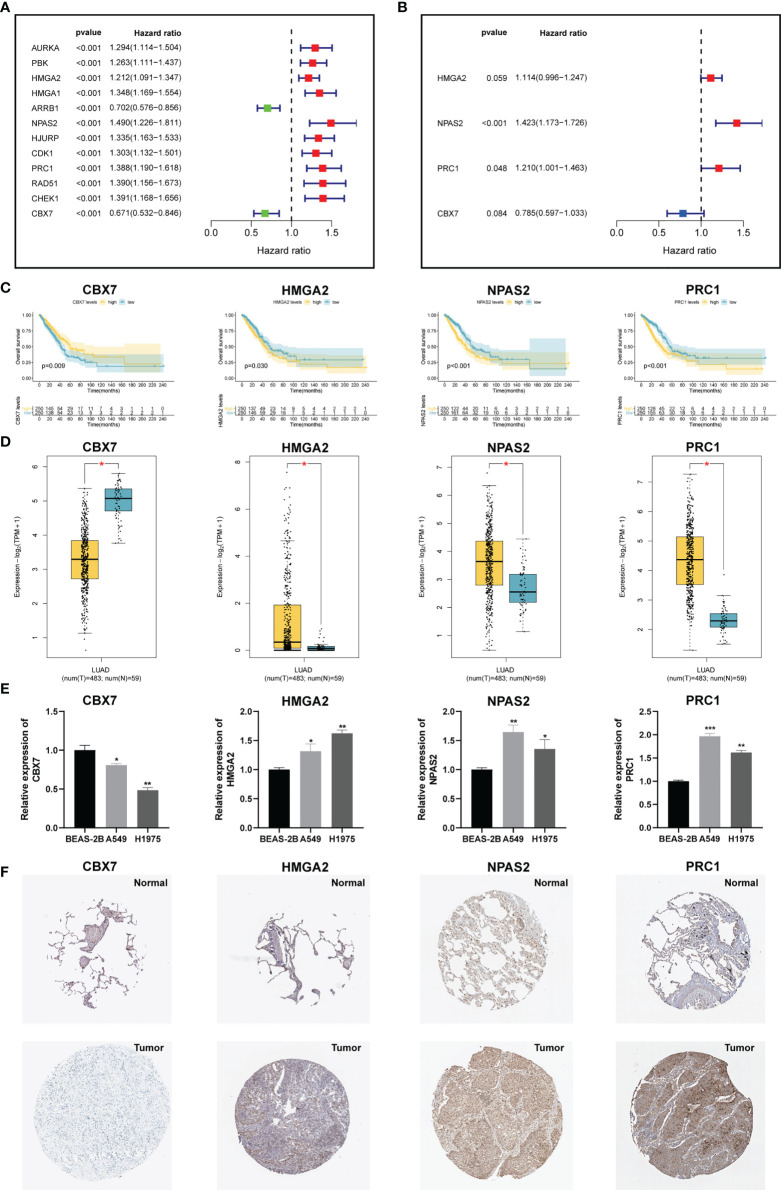
Construction of a CRs-based signature (CRBS). **(A, B)** Univariate and multivariate Cox analyses for signature establishment. **(C)** Survival analysis of four model CRs. **(D)** Comparison of differential expression of four model CRs based on GEPIA2 online portal. **(E)** The expression of four model CRs in BEAS-2B, A549 and HCI-H1975 cells line. **(F)** Immunohistochemistry of the CGs according to the HPA database. *p < 0.05; **p < 0.01; ***p < 0.001.

To develop an optimal prognostic signature, TCGA-LUAD cohort was selected as the training set. Univariate Cox regression was first employed to determine possible CRs with significant prognostic values (**Figure 3A**). Subsequently, 12 candidate genes were enrolled into multivariate Cox analysis to create a CRBS that included four risk CRs (**Figure 3B**). The risk formula was shown as follows: (0.1082 × HMGA2) + (0.3525 × NPAS2) + (0.1909 × PRC1) + (-0.2416 × CBX7). Survival curves illustrated that CBX7 was a potential favorable indicator, and HMGA2, NPAS2 and PRC1 were risky candidate indicators (**Figure 3C**). Then, we detect the expression differences of four CRs according to TCGA-LUAD dataset. All four CRs were greatly dysregulated between LUAD cases and control samples (**Figure 3D**). Furthermore, we validated the expression patterns of four model genes by qRT-PCR in cell lines. Consistent with the above bioinformatics analysis results, we noticed that CBX7 was downregulated in LUAD cell lines (A549 and HCI-H1975), and HMGA2, NPAS2 and PRC1 were overexpressed in LUAD cell lines compared to BEAS-2B (**Figure 3E**). Consistent with the above results, we detected the expression patterns of four CRs at IHC level based on HPA database (**Figure 3F**).

### Verification of the CRBS

**Figure 4:**
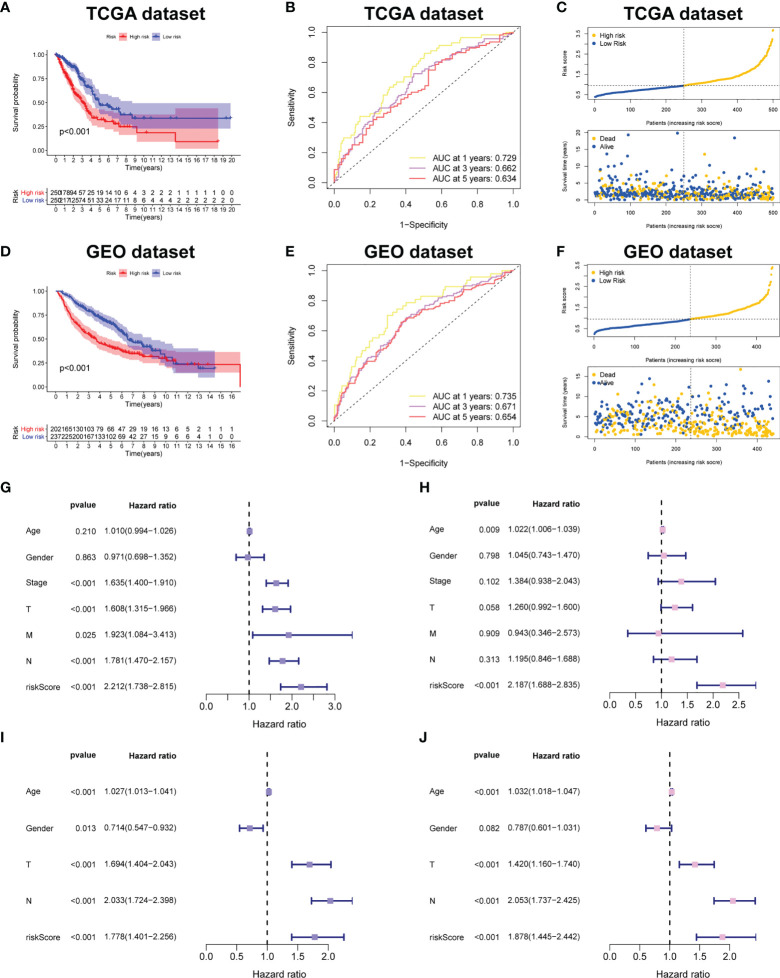
Prognostic powerful of the CRBS. **(A, D)** Survival analysis in the TCGA-LUAD and the GSE68465 cohorts. **(B, E)** ROC curves of the CRBS. **(C, F)** The risk distribution plots in two datasets. **(G–J)** Cox relevant regression assessing the independence of the CRBS.

**Figure 5:**
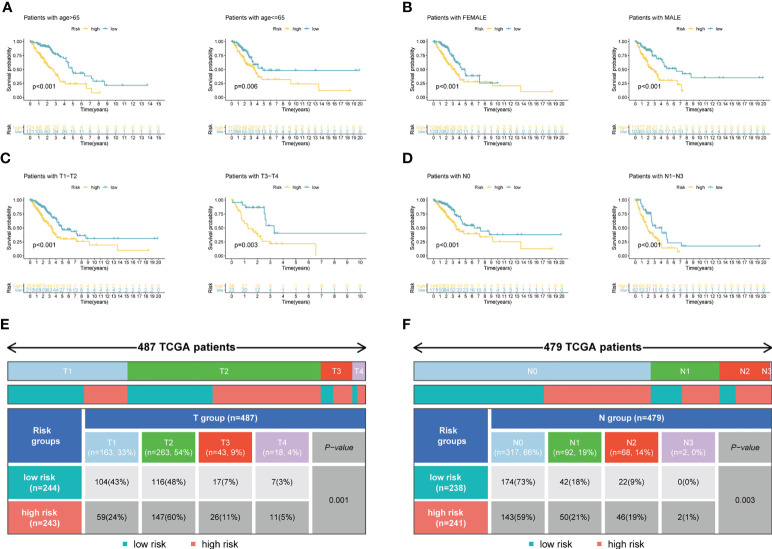
Subgroup survival analysis. **(A)** Age subgroup. **(B)** Gender subgroup. **(C)** T stage subgroup. **(D)** N stage subgroup. **(E, F)** Table presenting the distribution of T and N stage subgroups between two risk groups.

**Figure 4A** demonstrated that survival rates are lower in CRBS-high group compared to CRBS-low group in the training set. The AUC (area under the curve) values of 1-, 3-, and 5-year survival rates assessed by the CRBS were 0.729, 0.662, and 0.634, respectively (**Figure 4B**). **Figure 4C** summarizes the positive correlation between surviving cases and risk score. Moreover, we observed a similar trend of results in the test set, suggesting the favorable prediction ability of the CRBS (**Figures 4D–F**). To further unearth the independence of our model, univariate and multivariate Cox regression analyses were employed. Univariate analysis indicated that the risk score was an independent indicator for prognosis in both two datasets (**Figures 4G, I**). The multivariate method disclosed that risk score was independently associated with the dismal outcome of LUAD cases (**Figures 4H, J**). At the same time, we explore the performance of the CRBS based on a diversity of clinical subgroups. The results revealed that low risk score was correlated with favorable outcomes in different ages, genders, T stage and N stage cohorts (**Figures 5A–D**). Similarly, the good prediction capability of the CRBS was confirmed in the T stage and N stage subgroups (**Figures 5E, F**).

### Single-cell sequencing analysis

**Figure 6:**
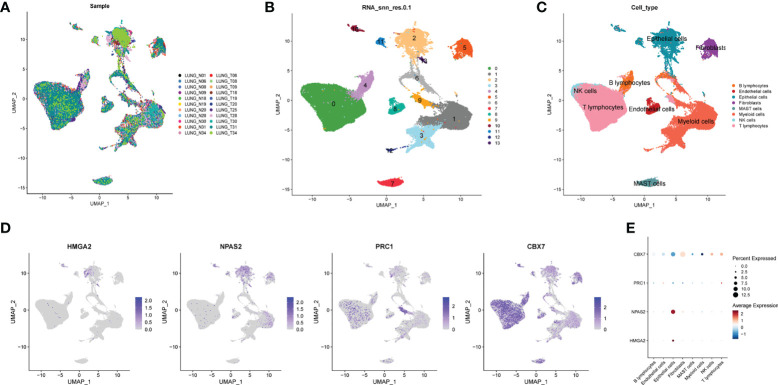
Single cell sequencing analysis. **(A)** The integration effect of 22 samples is favorable. **(B)** All cells in 22 samples were divided into 13 subgroups. **(C)** The cells were divided into 8 types of cell subgroups, namely B lymphocytes, endothelial cells, epithelial cells, fibroblasts, mast cells, myeloid cells, NK cells and T lymphocytes. **(D)** Cell location of four model CRs. **(E)** Correlation analysis of four model CRs and 8 types of cell subgroups.

To decipher the single-cell transcriptome dataset GSE131907, Seurat package was performed. The UMAP analysis suggested the distribution of the 22 LUAD samples (N = 11 and T = 11) with no remarkable batch effects (**Figure 6A**). All the cells were divided into 12 clusters the through k- Nearest Neighbor (KNN) clustering algorithm (**Figure 6B**). After performing cell annotation by different cell surface markers, we obtained eight cell subtypes, including B lymphocytes, endothelial cells, epithelial cells, fibroblasts, mast cells, myeloid cells, NK cells and T lymphocytes (**Figure 6C**). Next, we investigate the location of four CRs at single-cell transcriptome level. In **Figure 6D**,

HMGA2 and NPAS2 are mainly located in endothelial cells, and PRC1 and CBX7 are mainly located in NK cells and T lymphocytes. In addition, we noticed that the expression of CBX7 was negatively correlated with endothelial cells, whereas NPAS2 was positively correlated with endothelial cells (**Figure 6E**).

### GSEA determines CRBS-associated pathways

**Figure 7:**
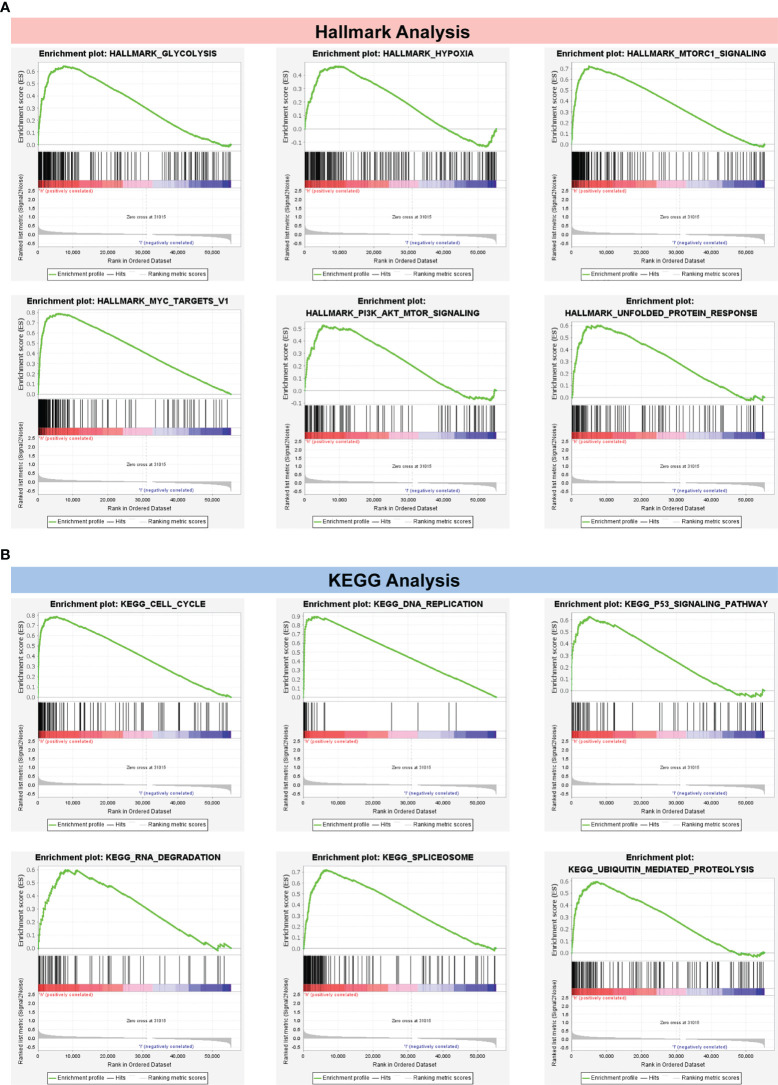
Gene Set Enrichment Analysis. **(A)** Hallmark analysis of the CRBS. **(B)** KEGG analysis of the CRBS.

In **Figure 7A**, the top six cancer hallmarks were remarkably enriched in the CRBS-high group, including glycolysis, hypoxia, mTORC1 pathway, MYC target, PI3K/Akt/mTOR and unfolded protein response. In terms of the KEGG analysis, we observed that CRBS-high group was involved in the cell cycle, p53 pathway and ubiquitination response (**Figure 7B**).

### Immune environment analysis

**Figure 8:**
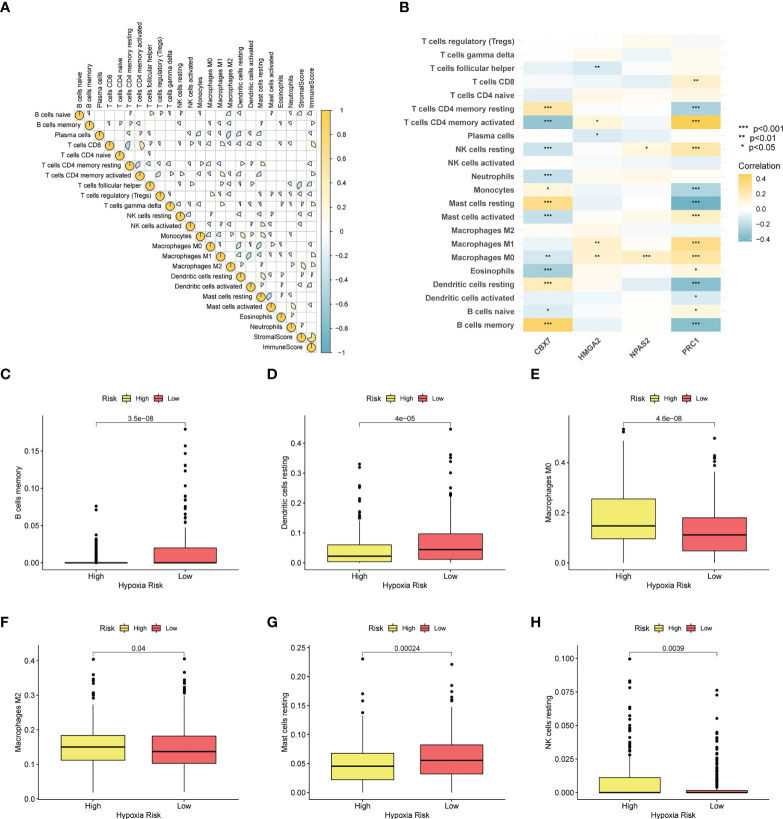
Immune infiltration analysis. **(A)** Correlation analysis of 22 immunocyte types. **(B)** Heatmap showing the relationship between four model CRs and immune cells. **(C–H)** The infiltration level differences of memory B cells, dendritic cells, macrophage M0, macrophage M2, mast cells and NK cells among two groups (*p< 0.05; **p< 0.01; ***p< 0.001).

To depict the immune landscape of LUAD, we evaluated the immunocyte infiltration of each case. **Figure 8A** summarizes the correlation between the 22 immunocyte types. As suggested in **Figure 8B**, CBX7 was greatly positively associated with memory B cells and resting mast cells. PRC1 was positively associated with activated memory T cells and negatively correlated with resting mast cells. Moreover, macrophage M0, macrophage M2 and resting NK cells were enriched in the CRBS-high group. Cases in CRBS-low group had greatly higher proportions of memory B cells, dendritic cells and mast cells (**Figures 8C–H**).

**Figure 9:**
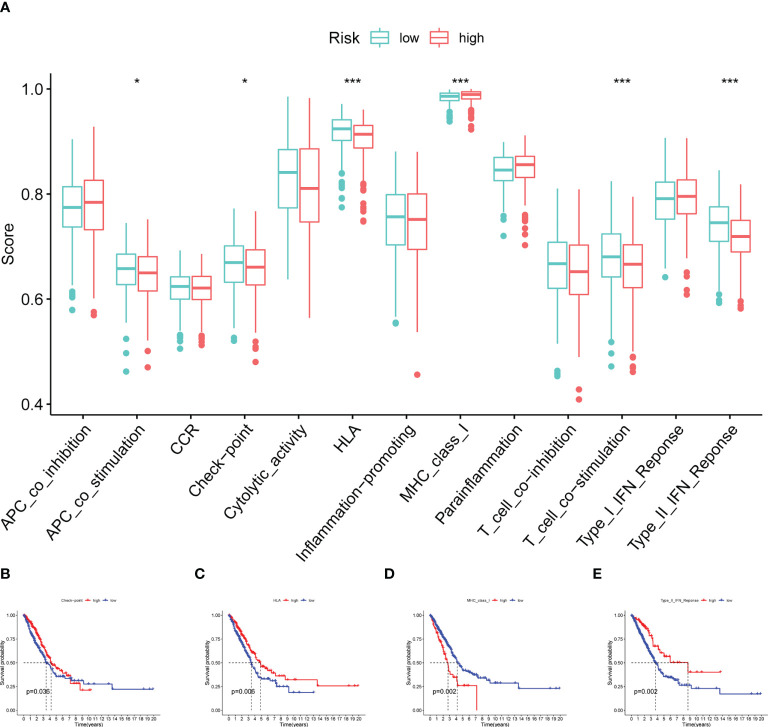
Immune function analysis. **(A)** Boxplot showing the relationship between four model CRs and 13 immune functions. **(B–E)** Survival analysis for checkpoint, HLA, MHC class I and type II IFN response (*p< 0.05; ***p< 0.001).

Additionally, some immune functions displayed differences between the two groups, including APC co-stimulation, checkpoint, HLA, MHC class I, T cell co-stimulation, and type II IFN response (**Figure 9A**). Also, we observed that four immune responses (checkpoint, HLA, MHC class I and type II IFN response) had significant differences in the outcome of patients with LUAD (**Figures 9B–E**).

### Clinical potency analysis of the CRBS

**Figure 10:**
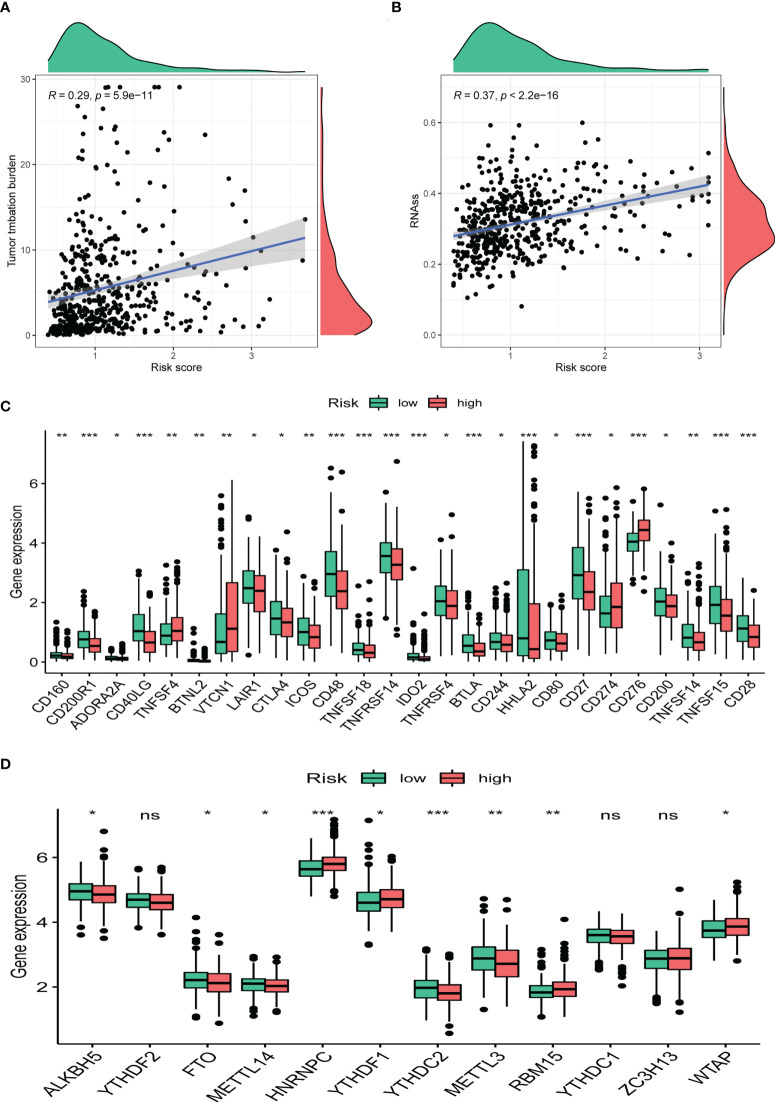
Clinical potency analysis. **(A)** TMB analysis of the CRBS. **(B)** Cancer stem cells index analysis the CRBS. Comparison of differential expression of **(C)** immune checkpoints and **(D)** m6A markers (*p< 0.05; **p< 0.01; ***p< 0.001). ns, no significance.

TMB has been demonstrated to be useful as an indicator of the efficacy of immunotherapy. We calculated the TMB of each LUAD sample and found that CRBS-high group had a higher TMB than the CRBS-low group (**Figures 10A**). Moreover, CRBS-high group presented a high level of mRNAsi (**Figure 10B**). In **Figure 10C**, most of the immune checkpoint markers were upregulated in the CRBS-high group. The comparison in the expression of m6A markers between the two groups indicated that the expression of ALKBH5, FTO, METTL14, HNRNPC, YTHDF1, YTHDF2, METTL3, RBM15 and WTAP were significant (**Figure 10D**).

## Discussion

LUAD is the most common pathological subtype of lung cancer, which is composed of approximately 40% of lung cancer cases (19). Despite the various efforts in improve, the five-year survival rate for LUAD patients remains shabby. Recent studies have suggested that patients with the same histology and TNM stages may have very distinct clinical outcomes, mainly due to their genetic heterogeneities (20). With the rapid development of the next-generation sequencing, a growing number of prognostic signatures based on transcriptome data were established to depict the individual differences, and to forecast the prognosis in various cancers (21–23). Therefore, a more reliable prognostic model based on genetic alterations is urgently needed to provide early detection and personalized treatment for LUAD patients.

It is well known that epigenetic alterations play a considerable role in mediating the tumor progression (24). As indispensable regulatory elements of epigenetics, CRs are involved in the onset and development of various cancer types including multiple myeloma, prostate cancer, hepatocellular carcinoma, and LUAD (25–28). In our current work, a total of four CRs including HMGA2, NPAS2, PRC1, and CBX7, were identified as effective prognostic biomarkers for predicting the prognosis of LUAD. Survival analysis indicated that HMGA2, NPAS2 and PRC1 are potential risky genes since their high expressions are correlated with dismal outcomes of LUAD samples, whereas CBX7 is candidate protective factor given that its high expression is associated with favorable outcomes of LUAD samples. The pro-tumor role of HMGA2 has been widely reported in multiple cancers including LUAD (29). HMGA2 was found highly expressed in the LUAD tissues compared with normal lung tissues, and HMGA2 silencing notably reduced the growth and metastasis of LUAD cell lines (30). In addition, a mechanistic study revealed that HMGA2 could induce epithelial-mesenchymal transition by activating MAPK/extracellular receptor kinase signaling in LUAD (31). Npas2 has been identified in peripheral tissues, possibly as a modulator of circadian rhythms (32). Qiu et al. once reported that in LUAD, the elevated expression level of NPAS2 is significantly related to poor prognosis (33). Conversely, it has been indicated that LUAD cases with low NPAS2 expression displayed a favorable clinical outcome by another team (34). Therefore, more basic researches are needed to elucidate the exact role of NPAS2 in LUAD. PRC1 has received widespread attention considering its diverse regulatory roles in a number of diseases, especially tumorigenesis (35). It has been suggested that overexpression of PRC1 triggers the onset of various cancers yet its potential roles in LUAD have not been fully understood (36). An ever-growing series of reports has demonstrated the aberrant expression of CBX7 in a variety of tumors (37). Mechanically, CBX7 may exert its tumor suppressor role by inhibiting the Wnt pathway and subsequently restrain the malignant character in LUAD (38).

GSEA unearthed that CRBS-high group were involved in glycolysis signaling, PI3K/AKT/mTOR signaling, and p53 signaling pathway using GSEA. Suppressed oxidative phosphorylation along with enhanced glycolysis, which is called the Warburg phenotype, is considered as metabolic marker of cancers (39). Vaupel et al. once reported that enhanced glycolysis accelerates lactic acid accumulation to impair the immune functions in TME and finally promote malignant progression (40). The PI3K/AKT/mTOR pathway plays a crucial role in diverse biological behaviors including cell growth, migration, metabolism, and death (41). In LUAD, the aberrant activation in this signaling has been indicated to induce uncontrolled growth, drug resistance, sustained angiogenesis, as well as distant metastasis (42). P53 protein is a transcription factor known as the “guardian gene” because of its significant role in preserving genomic integrity. The mutation of the p53 gene can be detected in a wide spectrum of human malignancies, including the breast, cervical, lung, and prostate cancer (43). More recently, Vokes and his colleagues provided the evidence that p53 alterations were involved in faster resistance evolution and may cooperate with other genomic events to gain resistance to EGFR tyrosine kinase inhibitors (44).

Immunotherapy that emerged recently has achieved promising results in the treatment of LUAD (45). In our work, a comprehensive analysis of tumor-infiltrating immune cells was further conducted to help to clarify the immune infiltration status between the two different risk groups. As a result, the infiltration level of HLA as well as the type 2 IFN was found downregulated remarkably in CRBS-high group. Also, the expression level of the immune checkpoint markers was validated to be correlated with the risk score. CD273, also named B3-H7, is overexpressed in various solid malignancies which serve as a potential therapeutic target (46, 47). Yu and his colleagues disclosed CD273 was upregulated in LUAD, and was correlated with lymph node metastasis (48). Likewise, accumulating studies have indicated the close association between the efficacy of immunotherapy and the CD274 expression (49). VTCN1, also named B7-H4, belongs to the co-stimulatory B7 family molecules and is associated with a poor prognosis in multiple cancer types (50–52). As revealed by a recent study, the elevation of VTCN1 expression is associated with LUAD with EGFR-activating mutations, which can ultimately cause resistance to immunotherapy in LUAD patients (53).

In view of the essential effect of m6A methylation modification in LUAD progression, we unearthed the expression patterns of m6A regulators between two risk groups. The results indicated that HNRNPC, YTHDF1, RBM15 and WTAP were enriched in the high-risk group. Lou and his colleagues demonstrated that YTHDF1 could facilitate LUAD growth and survival by enhancing Cyclin B1 translation (54). In addition, YTHDF1 has also been confirmed to have carcinogenic effects in many digestive system tumors including gastric cancer, hepatocellular carcinoma and colorectal cancer (55–57). Cheng et al. found that overexpression of WTAP correlate with dismal outcome of LC cases. In NSCLC, PCGEM1 could boost cancer cells proliferation by improving WTAP expression (58, 59).

There are still several limitations of the present study that need to be considered. Only expression data in gene level was analyzed to construct the prognostic model, and large-sample clinical data are still needed, as an external cohort, to evaluate the predictive value of our model. Additionally, although we have proven the reliable prognostic capacity of the four CR related genes, fundamental experiments are still needed to validate their precise functions in mediating LUAD progression.

## Conclusion

Taken together, our data may help provide opportunities for the development of new therapeutic strategies and elucidate the mechanism of tumor immune escape in LUAD. Our proposed model may usher in novel approaches to predicting prognosis of patients with LUAD.

## Data availability statement

The original contributions presented in the study are included in the article/**Supplementary Material**. Further inquiries can be directed to the corresponding author.

## Author contributions

QS and HM visualized the study and took part in the study design. QS, SH, XL, and SW performed the manuscript writing and bioinformatics analysis. All authors read and approved the final manuscript.

## Conflict of interest

The authors declare that the research was conducted in the absence of any commercial or financial relationships that could be construed as a potential conflict of interest.

## Publisher’s note

All claims expressed in this article are solely those of the authors and do not necessarily represent those of their affiliated organizations, or those of the publisher, the editors and the reviewers. Any product that may be evaluated in this article, or claim that may be made by its manufacturer, is not guaranteed or endorsed by the publisher.
