# Supplementary material for: Integrated single-cell and transcriptome sequencing analyses determines a chromatin regulator-based signature for evaluating prognosis in lung adenocarcinoma
Source: Front Oncol. 2022 Oct 17;12:1031728. doi: 10.3389/fonc.2022.1031728 (PMC9618736; doi:10.3389/fonc.2022.1031728)
Supplement: Supplementary file 1 [file Table_1.docx]

**Supplementary Table S1** The gene symbol of 870 CRs.

| A1CF |
| --- |
| ACTB |
| ACTL6A |
| ACTL6B |
| ACTR2 |
| ACTR3 |
| ACTR3B |
| ACTR5 |
| ACTR6 |
| ACTR8 |
| ADNP |
| AEBP2 |
| AHCTF1 |
| AICDA |
| AIRE |
| AKAP1 |
| ALKBH1 |
| ALKBH3 |
| ANP32A |
| ANP32B |
| ANP32E |
| APBB1 |
| APEX1 |
| APOBEC1 |
| APOBEC2 |
| APOBEC3A |
| APOBEC3B |
| APOBEC3C |
| APOBEC3D |
| APOBEC3F |
| APOBEC3G |
| APOBEC3H |
| ARID1A |
| ARID1B |
| ARID2 |
| ARID3A |
| ARID3B |
| ARID3C |
| ARID4A |
| ARID4B |
| ARID5A |
| ARID5B |
| ARNTL |
| ARRB1 |
| ASCL1 |
| ASCL2 |
| ASF1A |
| ASF1B |
| ASH1L |
| ASH2L |
| ASXL1 |
| ASXL2 |
| ASXL3 |
| ATAD2 |
| ATAD2B |
| ATF2 |
| ATF7IP |
| ATM |
| ATN1 |
| ATR |
| ATRX |
| ATXN7 |
| ATXN7L3 |
| AURKA |
| AURKB |
| AURKC |
| BABAM1 |
| BAG6 |
| BAHD1 |
| BANF1 |
| BANF2 |
| BANP |
| BAP1 |
| BARD1 |
| BAZ1A |
| BAZ1B |
| BAZ2A |
| BAZ2B |
| BBX |
| BCL10 |
| BCOR |
| BCORL1 |
| BMI1 |
| BOD1 |
| BPTF |
| BRCA1 |
| BRCA2 |
| BRCC3 |
| BRD1 |
| BRD2 |
| BRD3 |
| BRD4 |
| BRD7 |
| BRD8 |
| BRD9 |
| BRDT |
| BRE |
| BRMS1 |
| BRMS1L |
| BRPF1 |
| BRPF3 |
| BRWD1 |
| BRWD3 |
| BTAF1 |
| BUB1 |
| C14orf169 |
| C17orf49 |
| CARM1 |
| CBX1 |
| CBX2 |
| CBX3 |
| CBX4 |
| CBX5 |
| CBX6 |
| CBX7 |
| CBX8 |
| CDC6 |
| CDC73 |
| CDK1 |
| CDK17 |
| CDK2 |
| CDK3 |
| CDK5 |
| CDK7 |
| CDK9 |
| CDY1 |
| CDY2A |
| CDYL |
| CDYL2 |
| CECR2 |
| CENPC |
| CHAF1A |
| CHAF1B |
| CHD1 |
| CHD1L |
| CHD2 |
| CHD3 |
| CHD4 |
| CHD5 |
| CHD6 |
| CHD7 |
| CHD8 |
| CHD9 |
| CHEK1 |
| CHMP1A |
| CHMP1B |
| CHRAC1 |
| CHTOP |
| CHUK |
| CIC |
| CIR1 |
| CIT |
| CLNS1A |
| CLOCK |
| CRB2 |
| CREBBP |
| CSNK2A1 |
| CSRP2BP |
| CTBP1 |
| CTBP2 |
| CTCF |
| CTCFL |
| CTR9 |
| CUL1 |
| CUL2 |
| CUL3 |
| CUL4A |
| CUL4B |
| CUL5 |
| CXXC1 |
| DAPK3 |
| DAXX |
| DBF4 |
| DBF4B |
| DDB1 |
| DDB2 |
| DDX21 |
| DDX50 |
| DEK |
| DHX30 |
| DIDO1 |
| DMAP1 |
| DNAJC1 |
| DNAJC2 |
| DND1 |
| DNMT1 |
| DNMT3A |
| DNMT3B |
| DNMT3L |
| DNTT |
| DNTTIP2 |
| DOT1L |
| DPF1 |
| DPF2 |
| DPF3 |
| DPPA3 |
| DPY30 |
| DR1 |
| DTX3L |
| DUSP1 |
| DZIP3 |
| E2F6 |
| EED |
| EHMT1 |
| EHMT2 |
| EID1 |
| EID2 |
| EID2B |
| ELP2 |
| ELP3 |
| ELP4 |
| ELP5 |
| ELP6 |
| EMSY |
| ENY2 |
| EP300 |
| EP400 |
| EPC1 |
| EPC2 |
| ERBB4 |
| ERCC6 |
| ERCC6L |
| ERCC6L2 |
| EXOSC1 |
| EXOSC2 |
| EXOSC3 |
| EXOSC4 |
| EXOSC5 |
| EXOSC6 |
| EXOSC7 |
| EXOSC8 |
| EXOSC9 |
| EYA1 |
| EYA2 |
| EYA3 |
| EYA4 |
| EZH1 |
| EZH2 |
| FAM175A |
| FAM175B |
| FBL |
| FBRS |
| FBRSL1 |
| FBXL19 |
| FMR1 |
| FOXA1 |
| FOXO1 |
| FOXP1 |
| FOXP2 |
| FOXP3 |
| FOXP4 |
| FTO |
| FXR1 |
| FXR2 |
| GABRG1 |
| GADD45A |
| GADD45B |
| GADD45G |
| GATAD1 |
| GATAD2A |
| GATAD2B |
| GFI1 |
| GFI1B |
| GLYATL1 |
| GLYR1 |
| GSE1 |
| GSG2 |
| GTF2I |
| GTF3C1 |
| GTF3C4 |
| HAT1 |
| HBP1 |
| HCFC1 |
| HCFC2 |
| HDAC1 |
| HDAC10 |
| HDAC11 |
| HDAC2 |
| HDAC3 |
| HDAC4 |
| HDAC5 |
| HDAC6 |
| HDAC7 |
| HDAC8 |
| HDAC9 |
| HDGF |
| HELLS |
| HIF1AN |
| HINFP |
| HIRA |
| HIRIP3 |
| HJURP |
| HLCS |
| HLTF |
| HMG20A |
| HMG20B |
| HMGA1 |
| HMGA2 |
| HMGB1 |
| HMGB2 |
| HMGB3 |
| HMGN1 |
| HMGN2 |
| HMGN3 |
| HMGN4 |
| HMGN5 |
| HN1 |
| HN1L |
| HNF1A |
| HNRNPA1 |
| HP1BP3 |
| HR |
| HSPA1A |
| HUWE1 |
| IDH1 |
| IDH2 |
| IFIT3 |
| IGFBP7 |
| IKBKAP |
| IKZF1 |
| IKZF3 |
| ING1 |
| ING2 |
| ING3 |
| ING4 |
| ING5 |
| INO80 |
| INO80B |
| INO80C |
| INO80D |
| INO80E |
| JADE1 |
| JADE2 |
| JADE3 |
| JAK2 |
| JARID2 |
| JDP2 |
| JMJD1C |
| JMJD4 |
| JMJD6 |
| JMJD7 |
| JMJD8 |
| KANSL1 |
| KANSL2 |
| KANSL3 |
| KAT2A |
| KAT2B |
| KAT5 |
| KAT6A |
| KAT6B |
| KAT7 |
| KAT8 |
| KDM1A |
| KDM1B |
| KDM2A |
| KDM2B |
| KDM3A |
| KDM3B |
| KDM4A |
| KDM4B |
| KDM4C |
| KDM4D |
| KDM4E |
| KDM5A |
| KDM5B |
| KDM5C |
| KDM5D |
| KDM6A |
| KDM6B |
| KDM7A |
| KDM8 |
| KEAP1 |
| KMT2A |
| KMT2B |
| KMT2C |
| KMT2D |
| KMT2E |
| KMT5A |
| KMT5B |
| KMT5C |
| L3MBTL1 |
| L3MBTL2 |
| L3MBTL3 |
| L3MBTL4 |
| LAS1L |
| LBR |
| LEO1 |
| LMNA |
| LMNB1 |
| LMNB2 |
| LRWD1 |
| MAP3K7 |
| MAPKAPK3 |
| MASTL |
| MAX |
| MAZ |
| MBD1 |
| MBD2 |
| MBD3 |
| MBD4 |
| MBD5 |
| MBD6 |
| MBIP |
| MBTD1 |
| MCRS1 |
| MDC1 |
| MDM2 |
| MDM4 |
| MEAF6 |
| MECOM |
| MECP2 |
| MEN1 |
| MGA |
| MGEA5 |
| MGMT |
| MIER1 |
| MIER2 |
| MIER3 |
| MINA |
| MIS18BP1 |
| MLLT1 |
| MLLT10 |
| MLLT3 |
| MLLT6 |
| MOCS1 |
| MORF4 |
| MORF4L1 |
| MORF4L2 |
| MOV10 |
| MPHOSPH8 |
| MRGBP |
| MSH6 |
| MSL1 |
| MSL2 |
| MSL3 |
| MST1 |
| MTA1 |
| MTA2 |
| MTA3 |
| MTF2 |
| MUM1 |
| MYBBP1A |
| MYO1C |
| MYSM1 |
| NAA60 |
| NAP1L1 |
| NAP1L2 |
| NAP1L3 |
| NAP1L4 |
| NAP1L5 |
| NASP |
| NAT10 |
| NBN |
| NCL |
| NCOA1 |
| NCOA2 |
| NCOA3 |
| NCOA4 |
| NCOA5 |
| NCOA6 |
| NCOA7 |
| NCOR1 |
| NCOR2 |
| NEK6 |
| NEK9 |
| NFRKB |
| NFYB |
| NFYC |
| NIPBL |
| NOC2L |
| NPAS2 |
| NPM1 |
| NPM2 |
| NSD1 |
| NSL1 |
| OGT |
| ORC1 |
| ORC2 |
| PADI1 |
| PADI2 |
| PADI3 |
| PADI4 |
| PAF1 |
| PAGR1 |
| PAK2 |
| PARG |
| PARP1 |
| PARP2 |
| PARP3 |
| PARP4 |
| PATZ1 |
| PAXIP1 |
| PBK |
| PBRM1 |
| PCGF1 |
| PCGF2 |
| PCGF3 |
| PCGF5 |
| PCGF6 |
| PCNA |
| PDP1 |
| PDS5A |
| PDS5B |
| PELP1 |
| PES1 |
| PHC1 |
| PHC2 |
| PHC3 |
| PHF1 |
| PHF10 |
| PHF12 |
| PHF13 |
| PHF14 |
| PHF19 |
| PHF2 |
| PHF20 |
| PHF20L1 |
| PHF21A |
| PHF21B |
| PHF23 |
| PHF3 |
| PHF6 |
| PHF7 |
| PHF8 |
| PHIP |
| PIWIL4 |
| PKM |
| PKN1 |
| POGZ |
| POLE3 |
| PPARGC1A |
| PPM1G |
| PPP2CA |
| PPP4C |
| PPP4R2 |
| PPP4R3A |
| PPP4R3B |
| PPP4R3CP |
| PRC1 |
| PRDM1 |
| PRDM10 |
| PRDM11 |
| PRDM12 |
| PRDM13 |
| PRDM14 |
| PRDM15 |
| PRDM16 |
| PRDM2 |
| PRDM4 |
| PRDM5 |
| PRDM6 |
| PRDM7 |
| PRDM8 |
| PRDM9 |
| PRKAA1 |
| PRKAA2 |
| PRKAB1 |
| PRKAB2 |
| PRKAG1 |
| PRKAG2 |
| PRKAG3 |
| PRKCA |
| PRKCB |
| PRKCD |
| PRKDC |
| PRMT1 |
| PRMT2 |
| PRMT3 |
| PRMT5 |
| PRMT6 |
| PRMT7 |
| PRMT8 |
| PRMT9 |
| PRPF31 |
| PRR12 |
| PRR14 |
| PSIP1 |
| PWWP2B |
| PYGO1 |
| PYGO2 |
| RAC3 |
| RAD51 |
| RAD54B |
| RAD54L |
| RAD54L2 |
| RAG1 |
| RAG2 |
| RAI1 |
| RARA |
| RB1 |
| RBBP4 |
| RBBP5 |
| RBBP7 |
| RBP1 |
| RBX1 |
| RCC1 |
| RCOR1 |
| RCOR2 |
| RCOR3 |
| REST |
| REV1 |
| RING1 |
| RIT1 |
| RLIM |
| RMI1 |
| RNF168 |
| RNF17 |
| RNF2 |
| RNF20 |
| RNF40 |
| RNF8 |
| RPS6KA3 |
| RPS6KA4 |
| RPS6KA5 |
| RRP8 |
| RSAD1 |
| RSF1 |
| RTF1 |
| RUVBL1 |
| RUVBL2 |
| RYBP |
| SAFB |
| SAP130 |
| SAP18 |
| SAP25 |
| SAP30 |
| SAP30L |
| SATB1 |
| SATB2 |
| SCMH1 |
| SCML1 |
| SCML2 |
| SCML4 |
| SENP1 |
| SENP3 |
| SET |
| SETBP1 |
| SETD1A |
| SETD1B |
| SETD2 |
| SETD3 |
| SETD4 |
| SETD5 |
| SETD6 |
| SETD7 |
| SETD8P1 |
| SETD9 |
| SETDB1 |
| SETDB2 |
| SETMAR |
| SF3B1 |
| SF3B3 |
| SFMBT1 |
| SFMBT2 |
| SFPQ |
| SGF29 |
| SHPRH |
| SIN3A |
| SIN3B |
| SIRT1 |
| SIRT2 |
| SIRT3 |
| SIRT4 |
| SIRT5 |
| SIRT6 |
| SIRT7 |
| SKP1 |
| SLF1 |
| SMARCA1 |
| SMARCA2 |
| SMARCA4 |
| SMARCA5 |
| SMARCAD1 |
| SMARCAL1 |
| SMARCB1 |
| SMARCC1 |
| SMARCC2 |
| SMARCD1 |
| SMARCD2 |
| SMARCD3 |
| SMARCE1 |
| SMC1A |
| SMCHD1 |
| SMYD1 |
| SMYD2 |
| SMYD3 |
| SMYD4 |
| SMYD5 |
| SNAI2 |
| SND1 |
| SP1 |
| SP100 |
| SP110 |
| SP140 |
| SPEN |
| SPOP |
| SRCAP |
| SRRM2 |
| SRSF1 |
| SRSF3 |
| SS18L1 |
| SS18L2 |
| SSRP1 |
| STK31 |
| STK4 |
| SUDS3 |
| SUPT16H |
| SUPT3H |
| SUPT6H |
| SUPT7L |
| SUV39H1 |
| SUV39H2 |
| SUZ12 |
| SYNCRIP |
| TADA1 |
| TADA2A |
| TADA2B |
| TADA3 |
| TAF1 |
| TAF10 |
| TAF12 |
| TAF1L |
| TAF2 |
| TAF3 |
| TAF4 |
| TAF5 |
| TAF5L |
| TAF6 |
| TAF6L |
| TAF7 |
| TAF8 |
| TAF9 |
| TAF9B |
| TBL1XR1 |
| TCF4 |
| TDG |
| TDRD1 |
| TDRD12 |
| TDRD3 |
| TDRD5 |
| TDRD7 |
| TDRD9 |
| TDRKH |
| TERF1 |
| TERF2 |
| TET1 |
| TET2 |
| TET3 |
| TEX10 |
| TFDP1 |
| TFF1 |
| TFPT |
| TLE1 |
| TLE2 |
| TLE4 |
| TLK1 |
| TLK2 |
| TNP1 |
| TNP2 |
| TONSL |
| TOP2A |
| TOP2B |
| TOPBP1 |
| TOX |
| TOX2 |
| TOX3 |
| TOX4 |
| TP53 |
| TP53BP1 |
| TRDMT1 |
| TRIM16 |
| TRIM24 |
| TRIM27 |
| TRIM28 |
| TRIM33 |
| TRRAP |
| TSPY1 |
| TSPYL1 |
| TSPYL2 |
| TSPYL4 |
| TSPYL5 |
| TSPYL6 |
| TSSK6 |
| TTF2 |
| TTK |
| TXN2 |
| TYW5 |
| UBE2A |
| UBE2B |
| UBE2D1 |
| UBE2D3 |
| UBE2E1 |
| UBE2H |
| UBE2N |
| UBE2T |
| UBN1 |
| UBR2 |
| UBR5 |
| UBR7 |
| UBTF |
| UCHL5 |
| UHRF1 |
| UHRF2 |
| UIMC1 |
| UNK |
| USP11 |
| USP12 |
| USP15 |
| USP16 |
| USP17L2 |
| USP21 |
| USP22 |
| USP3 |
| USP36 |
| USP44 |
| USP46 |
| USP49 |
| USP7 |
| UTY |
| VDR |
| VEZF1 |
| VPS72 |
| VRK1 |
| WAC |
| WDR5 |
| WDR77 |
| WDR82 |
| WHSC1 |
| WHSC1L1 |
| WSB2 |
| XRCC1 |
| YAF2 |
| YEATS2 |
| YEATS4 |
| YWHAB |
| YWHAE |
| YWHAZ |
| YY1 |
| ZBTB16 |
| ZBTB24 |
| ZBTB33 |
| ZBTB38 |
| ZBTB4 |
| ZBTB7C |
| ZCWPW1 |
| ZCWPW2 |
| ZFAT |
| ZFP57 |
| ZGPAT |
| ZHX1 |
| ZHX2 |
| ZHX3 |
| ZMYM1 |
| ZMYM2 |
| ZMYM3 |
| ZMYM4 |
| ZMYM5 |
| ZMYM6 |
| ZMYND11 |
| ZMYND8 |
| ZNF217 |
| ZNF516 |
| ZNF532 |
| ZNF541 |
| ZNF592 |
| ZNF687 |
| ZNF711 |
| ZNHIT1 |
| ZRANB3 |
| ZZZ3 |
