# Supplementary material for: Integrated single-cell and transcriptome sequencing analyses determines a chromatin regulator-based signature for evaluating prognosis in lung adenocarcinoma
Source: Front Oncol. 2022 Oct 17;12:1031728. doi: 10.3389/fonc.2022.1031728 (PMC9618736; doi:10.3389/fonc.2022.1031728)
Supplement: Supplementary file 2 [file Table_2.docx]

**Supplementary Table S2** Primer sequences for each gene.

| Gene | Primer sequence | |
| --- | --- | --- |
| GAPDH | Forward | 5′-CTGGGCTACACTGAGCACC-3′ |
|  | Reverse | 5′-AAGTGGTCGTTGAGGGCAATG-3′ |
| CBX7 | Forward | 5′-GCGTGCGGAAGGGTAAAGT-3′ |
|  | Reverse | 5′-GCTTGGGTTTCGGACCTCTC-3′ |
| HMGA2 | Forward | 5′-ACCCAGGGGAAGACCCAAA-3′ |
|  | Reverse | 5′-CCTCTTGGCCGTTTTTCTCCA-3′ |
| NPAS2 | Forward | 5′-CGTGTTGGAAAAGGTCATCGG-3′ |
|  | Reverse | 5′-TCCAGTCTTGCTGAATGTCAC-3′ |
| PRC1 | Forward | 5′-ATCACCTTCGGGAAATATGGGA-3′ |
|  | Reverse | 5′-TCTTTCTGACAGACGGATATGCT-3′ |
